# Morphologic and Microvascular Differences Between Macular Neovascularization With and Without Subretinal Fibrosis

**DOI:** 10.1167/tvst.10.14.1

**Published:** 2021-12-01

**Authors:** Philipp Ken Roberts, Markus Schranz, Alice Motschi, Sylvia Desissaire, Valentin Hacker, Michael Pircher, Stefan Sacu, Wolf Buehl, Christoph Konrad Hitzenberger, Ursula Schmidt-Erfurth

**Affiliations:** 1 Department of Ophthalmology and Optometry of the Medical University of Vienna, Vienna, Austria; 2 Center for Medical Physics and Biomedical Engineering of the Medical University of Vienna, Vienna, Austria

**Keywords:** age-related macular degeneration, optical coherence tomography, optical coherence tomography angiography

## Abstract

**Purpose:**

To evaluate morphologic and microvascular differences between eyes with and without subretinal fibrosis (SF) caused by neovascular age-related macular degeneration (nAMD).

**Methods:**

Patients with nAMD with a minimum history of 12 months of anti-VEGF treatment were prospectively included in this cross-sectional study. Patients were imaged using standard imaging, swept-source optical coherence tomography angiography for quantitative microvascular analysis and polarization-sensitive OCT as an ancillary method for automated SF segmentation. The presence of reticular pseudodrusen, hyperreflective foci (HRF), and outer retinal tubulation (ORT) were also evaluated.

**Results:**

Sixty eyes of 60 participants (37 female) with nAMD and a mean 3.1 (±2.7)-year history of anti-VEGF treatment were included, 20 (33%) of which were diagnosed with SF. Eyes with SF had a higher prevalence of ORT (*P* < 0.001) and a lower prevalence of HRF (*P* = 0.004) than eyes without SF. Fifty eyes were analyzed quantitatively for microvascular biomarkers. Eyes with SF had a larger greatest vascular caliber (*P* = 0.001) and greatest linear diameter (*P* = 0.042), a larger microvascular neovascularization (MNV) area (*P* = 0.026), larger vessel area (*P* = 0.037), higher number of vessel junctions (*P* = 0.025), longer total vessel length (*P* = 0.027), higher number of vessel endpoints (*P* = 0.007), and higher endpoint density (*P* = 0.047).

**Conclusions:**

This multimodal imaging approach demonstrated in vivo microvascular and morphological differences in eyes with and without SF. Eyes with SF tend to have larger MNV lesions with thicker vessels and are often associated with the presence of ORT.

**Translational Relevance:**

This study points out imaging biomarkers in patients with SF, which may help identifying high-risk patients.

## Introduction

Long-term follow-up of patients with neovascular age-related macular degeneration (nAMD) revealed the presence of subretinal fibrosis (SF) in 32% to 36% after one year, 40% to 45% after two years, up to 61% after seven years, and in up to 71% after 10 years despite regular control visits and standardized antivascular endothelial growth factor (VEGF) treatment.^1^^–^^7^ SF causes irreversible damage to the retina and, together with macular atrophy, is the major culprit in nAMD with poor visual outcome.^1^^,^^7^^–^^9^ Thus prevention of fibrosis and novel antifibrotic therapies are in the focus of researchers worldwide.^10^^–^^12^ Clearly defined endpoints for interventional trials, however, are lacking because detection and quantification of SF is challenging using standard retinal imaging modalities. Clinical staging of fibrosis is subjective and current gold standard imaging such as color fundus photography (CFP) and optical coherence tomography (OCT), even in concert, often do not allow a distinct and early delineation of SF. Novel imaging modalities such as polarization-sensitive OCT (PS-OCT) and OCT angiography (OCTA) are promising means to objectively detect fibrosis and provide insights into microvascular and neurosensory compartments.^13^^–^^17^

PS-OCT detects ocular structures such as fibrosis or the retinal pigment epithelium (RPE) based on tissue-specific contrast and facilitates differentiation of macular neovascularization (MNV) components in vivo.^17^^,^^18^ Additionally, PS-OCT has advantages in the detection of retinal morphological features associated with nAMD such as reticular pseudodrusen (RPD) or intraretinal RPE migration, also referred to as hyperreflective foci (HRF).^19^^,^^20^ Outer retinal tubulation (ORT), a typical retinal morphological finding associated with advanced AMD can also be readily detected by PS-OCT based on intensity imaging.^21^

OCTA, an extension of conventional OCT, offers noninvasive and automated detection of blood vessels and can be used to analyze the microvasculature of MNV lesions in great detail.^14^^,^^22^ Using validated and open-source analysis software, quantitative evaluation of vascular features such as vessel area, total vessel length, number of vessel junctions within the lesion, number of vessel endpoints or lacunarity can be performed, providing objective and detailed information about the microvascular configuration beyond qualitative descriptions of morphologic patterns such as “dead tree” or “sea fan” shape.^23^^–^^27^

Choi et al.^24^ recently used quantitative OCTA image analysis to differentiate eyes with nAMD and type 1 MNV on “stable” versus “unstable” treatment intervals. Querques et al.^28^ used quantitative OCTA parameters such as perfusion density to assess differences between fibrocellular and fibrovascular phenotypes of nAMD in remission. They detected preserved neovascular complexes with a reduced perfusion density in eyes of the fibrocellular type.

The purpose of this study was to evaluate microvascular and outer retinal differences between eyes with nAMD with SF compared to eyes without SF using multimodal imaging including OCTA and PS-OCT.

## Methods

### Study Design and Participants

This was a cross-sectional study conducted at the Department of Ophthalmology and Optometry and Center for Medical Physics and Biomedical Engineering (CMPBE) at the Medical University of Vienna. The study protocol (NCT03838679) and procedures adhered to the tenets of the Declaration of Helsinki and were approved by the ethics committee of the Medical University of Vienna. Informed consent was obtained from each participant before inclusion.

Sixty eyes of 60 patients with nAMD and a minimum history of 12 months of anti-VEGF therapy were consecutively enrolled and examined according to a standardized protocol. Patients with media opacification preventing high-quality imaging or retinal disease other than AMD were not included in the study. All patients had a full ophthalmic examination including best-corrected visual acuity testing using the early treatment of diabetic retinopathy study (ETDRS) score, slit lamp and dilated fundus examination. and SD-OCT, as well as fluorescein angiography (FA) and indocyanine green (ICGA) imaging using the Spectralis HRA+OCT device (Heidelberg Engineering, Heidelberg, Germany). CFP imaging was performed using the integrated color fundus camera of the Micro Perimeter 3 (Nidek Tokyo, Japan) device, which achieves high-quality, true-color images.

### MNV Grading and SF Identification

Neovascularization was classified as type 1 (= sub-RPE), type 2 (= subretinal), mixed type (subretinal + sub-RPE), type 3 (= retinal angiomatous proliferation) or polypoidal choroidal vasculopathy, based on FA, ICGA and SD-OCT imaging.^29^ The presence of SF was determined as either present or not present on CFP imaging and PS-OCT. On CFP SF was defined as whitish or yellowish material not related to drusen, hard exudate, fibrin, or dehemoglobinized blood associated with hyperreflective material on SD-OCT imaging^18^ and identified by two independent reading center–certified graders (P.K.R., M.S.). In ambiguous cases, open adjudication between the two graders was performed to reach consensus. On PS-OCT SF was detected automatically by a proprietary algorithm based on tissue birefringence.

### OCTA Imaging and Image Analysis

Patients were imaged with a swept-source optical coherence tomography angiography (OCTA) device (Plex Elite 9000; Carl Zeiss Meditec, Dublin, CA, USA) with an integrated eye-tracker using a 6 × 6 mm scan pattern centered on the fovea. The system operates at a center wavelength of 1060 nm, allowing deeper penetration into tissue than systems operating at shorter wavelengths, which may be of particular value in areas of SF.

Only eyes with a well-centered volume scan, and minimum signal strength of seven (of 10) were included in the study. Eyes without a clearly detectable MNV were excluded from the quantitative OCTA evaluation and were only evaluated for MNV type and structural outer retinal findings based on FA, ICGA and SD-OCT imaging.

Of the 60 eyes enrolled in the study, 50 eyes could be evaluated quantitatively by OCTA. Nine eyes without SF did not show a clearly defined MNV network on OCTA and were excluded from the quantitative OCTA analysis. One eye with SF was excluded from quantitative OCTA analysis because of severe blinking and motion artifacts.

To achieve en face OCTA images with the best possible display of the MNV, the anterior border of the slab was aligned with the anterior border of the MNV complex, and the posterior border of the slab was aligned with Bruch's membrane using the built-in software of the Plex Elite 9000 device. Segmentation errors were adjusted manually. The integrated projection artifact removal function was used to eliminate artifacts originating from the retinal vasculature.

**Figure 1.**
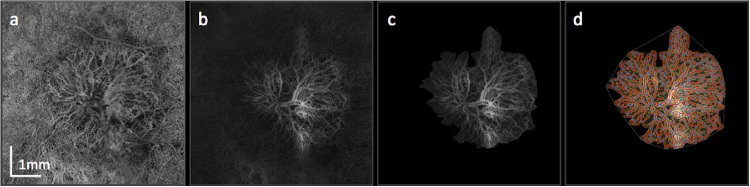
Post processing steps for quantitative microvascular analysis using optical coherence tomography angiography. The initial choriocapillaris en face map (a) does not show a clear border between the MNV and the surrounding choriocapillaris network. An individualized slab was chosen for every MNV lesion (b) with the anterior border aligned with the anterior border of the MNV complex and the posterior border aligned with Bruch's membrane. After manual delineation (c) of the MNV complex and Angiotool analysis, the program displays the vessels (highlighted in red) and junctions (highlighted as blue dots) as an overlay (d).

For quantitative analysis of the vascular components of the MNV complex we used the publicly available image processing software FIJI (ImageJ 2.0.0, imagej.net) and the open-source and validated software Angiotool (version 0.6a, https://ccrod.cancer.gov/confluence/display/ROB2/Downloads) (Fig. 1).^24^^,^^26^^,^^27^^,^^30^^–^^33^

After manual delineation of the MNV complex, the greatest linear diameter (GLD) was measured in micrometers as the greatest distance enclosed by the MNV area.^25^ The greatest vascular caliber (GVC) was measured in micrometers in FIJI with magnification ×300 using the measurement tool as described previously.^23^^,^^24^ In the Angiotool software, the parameters were optimized as described previously.^26^ The low threshold was set between 9 and 35 depending on intensity of the flow signal, and the high threshold was set to 255, vessel thickness was set between 3 and 6 depending on vessel calibers within the MNV lesion, and removal of small particles was set between 10 and 80. The program then automatically measures the lesion area, vessel area (equivalent to the area with flow signal within the MNV complex), number of vessel junctions within the MNV complex, total vessel length (sum of all vessels), the total number of endpoints, as well as lacunarity, reflecting the inhomogeneity of the neovascular structure.^26^ The junction density per unit vessel length was calculated as total number of junctions/total vessel length as described previously.^34^ Vessel length density (total length of vessel/ vessel area), junction density (total number of vessel junctions/total length of vessel) and endpoint density (total number of vessel endpoints/total length of vessel) were calculated as described previously.^24^

### PS-OCT Imaging and Image Analysis

For PS-OCT imaging we used a custom-built SD-OCT based PS-OCT device developed at the CMPBE incorporating a line-scanning laser ophthalmoscopy channel for real-time retinal tracking.^35^ The system operates at a center wavelength of 863 nm with a full-width half maximum bandwidth of 60 nm and acquires volume scans of 8 × 6 mm containing 250 B-scans with 1,024 A-scans in 4.5 seconds. The specifications of the device have been described in detail.^35^ In brief, the probing light beam is fed into a Michelson interferometer using polarization-maintaining fiber optics. Circularly polarized light is used for retinal illumination and the backscattered light is detected by a polarization-sensitive detection unit consisting of a polarizing beam splitter and two identical spectrometers, one for each of the two orthogonal polarization-channels.

**Figure 2.**
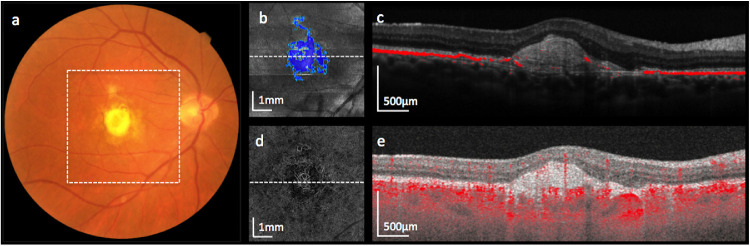
Right eye of a patient with type 2 MNV and subretinal fibrosis. Color fundus photography (a), polarization-sensitive optical coherence tomography (PS-OCT) en face intensity image with segmented subretinal fibrosis shown in blue (b), PS-OCT intensity B-scan with segmented retinal pigment epithelium in red (c), OCTA en face image showing the MNV complex (d) and OCTA B-scan with flow highlighted in red (e) are presented. The location of the en face images is highlighted by a white dotted square in (a) and the location of B-scans is highlighted by dotted white lines in (b) and (d). The yellow-whitish material in (a), clinically diagnosed as subretinal fibrosis, overlaps with the area of automatically segmented fibrosis in PS-OCT (b).

Different images (reflectivity, phase retardation, optic axis orientation, degree of polarization uniformity) are computed from PS-OCT raw data and used for selective tissue imaging. Phase retardation and optic axis orientation are used to detect birefringent tissue such as fibrosis, whereas degree of polarization uniformity is used for automatic detection of RPE based on the polarization-scrambling effect of melanin within RPE cells.^36^^,^^37^ For detection of SF we applied an algorithm that improves on our previous work^17^ by taking advantage of the optic axis uniformity, a parameter that has higher values in fibrotic tissue and low values in surrounding non-birefringent tissue (Fig. 2).^38^ Please find details of the algorithm in a recent publication.^39^

Furthermore, PS-OCT images were graded for the presence of RPD, HRF, and ORT based on PS-OCT B-scans (P.K.R., M.S.). RPD were defined as deposition of granular hyperreflective material between the RPE and the inner and outer segment junction of photoreceptors (IS/OS) typically associated with thinning of the RPE.^19^^,^^40^ HRF were defined as depolarizing, hyperreflective dots within the neurosensory retina.^41^ ORT was defined as ovoid or round hyperreflective structure in the outer nuclear layer with a relatively hyporeflective center.^42^

### Statistical Analysis

Statistical analyses were performed using IBM SPSS statistics version 21 (IBM SPSS Statistics; IBM Corporation, Chicago, IL, USA). Descriptive statistics and Fisher's exact test were used for qualitative findings. Independent samples Mann-Whitney U test was used to assess differences of quantitative parameters. No adjustment for multiple testing was performed as the goals of the study are exploratory rather than confirmatory. A *P* value <0.05 was considered statistically significant.

## Results

Sixty eyes of 60 consecutive patients (37 female) with nAMD and a mean 3.1 (±2.7) year history of anti-VEGF treatment with 14.6 (±10.0) intravitreal injections were included in this cross-sectional study. Mean age was 77.4 (±6.2) years, and best-corrected visual acuity (LogMAR) was 0.45 (±0.44) with an ETDRS letter score of 63 (±22), respectively. Twenty eyes (33%) were diagnosed with SF based on CFP in combination with PS-OCT. Detailed characteristics of patients and MNV features are presented in Table 1. There was no difference in age, gender, number of anti-VEGF injections, or time since diagnosis of nAMD between the SF group and the group without SF (*P* > 0.05). Eyes in the non-SF group had a higher median (minimum-maximum) ETDRS letter score than eyes in the SF group (78 [25–90] versus 39 [6–79]; *P* < 0.001) and a lower LogMAR (0.2 [−0.1 to 1.1] versus 0.9 [0.1 to 1.6]; *P* < 0.001).

**Table 1.** Characteristics of Study Participants and Macular Neovascularization Features

	Subretinal Fibrosis	No Subretinal	
Patient Characteristics	(n = 20)	Fibrosis (n = 40)	*P* Value
Age (years), median (min-max)	77.0 (62.2–87.3)	77.5 (59.6–87.0)	0.546
BCVA (ETDRS-Score), median (min-max)	39 (6–79)	78 (25–90)	<0.001^*^
BCVA (LogMAR), median (min-max)	0.9 (0.1–1.6)	0.2 (−0.1–1.1)	<0.001^*^
Time since first injection (years), median (min-max)	2.2 (1.0–10.8)	1.4 (1.0–10.8)	0.331
Number of intravitreal anti-VEGF injections median (min-max)	12 (7–43)	10 (3–46)	0.368
Gender (f)	14 (70%)	23 (58%)	0.408
Reticular pseudodrusen	7 (35%)	18 (45%)	0.581
Hyperreflective foci	7 (35%)	30 (75%)	0.004^*^
Outer retinal tubulation	17 (85%)	4 (10%)	<0.001^*^
MNV types			
Type 1 MNV	5 (25%)	29 (73%)	<0.001^*^
Type 2 MNV	5 (25%)	1 (3%)	0.013^*^
RAP	1 (5%)	7 (18%)	0.249
PCV	2 (10%)	2 (5%)	0.595
Mixed (type 1+2)	7 (35%)	1 (3%)	0.001^*^
Subretinal MNV component	12 (60%)	2 (5%)	<0.001^*^

BCVA, best-corrected visual acuity; LogMAR, logarithm of minimal angle of resolution; RAP, retinal angiomatous proliferation; PCV, polypoidal choroidal vasculopathy.

* Statistically significant *P* values.

Mixed type MNV was the most common type in the SF group (n = 7, 35%) and had a higher prevalence than in the non-SF group (n = 1, 3%; *P* = 0.001). Type 1 MNV was the most common type in eyes without SF (n = 29, 73%) and had a higher prevalence than in eyes with SF (n = 5, 25%; *P* < 0.001). When combining all eyes with a subretinal MNV component (mixed type + type 2 MNV), there was a higher prevalence in the SF group (n = 12, 60%) than in the non-SF group (n = 2, 5%; *P* < 0.001). The MNV membrane involved the subfoveal area in all eyes. Similarly, the area of SF involved the subfoveal area in all eyes except for one eye with polypoidal choroidal vasculopathy, where SF was observed extrafoveally.

### Morphological Analysis

ORT was observed more often in eyes with SF (n = 17, 85%) than in eyes without SF (n = 4, 10%; *P* < 0.001) and HRF were observed more often in the non-SF group (n = 30, 75%) than in the SF group (n = 7, 35%; *P* = 0.004). No significant difference was observed in the number of eyes with RPD.

### Quantitative Analysis of OCTA Imaging

**Figure 3.**
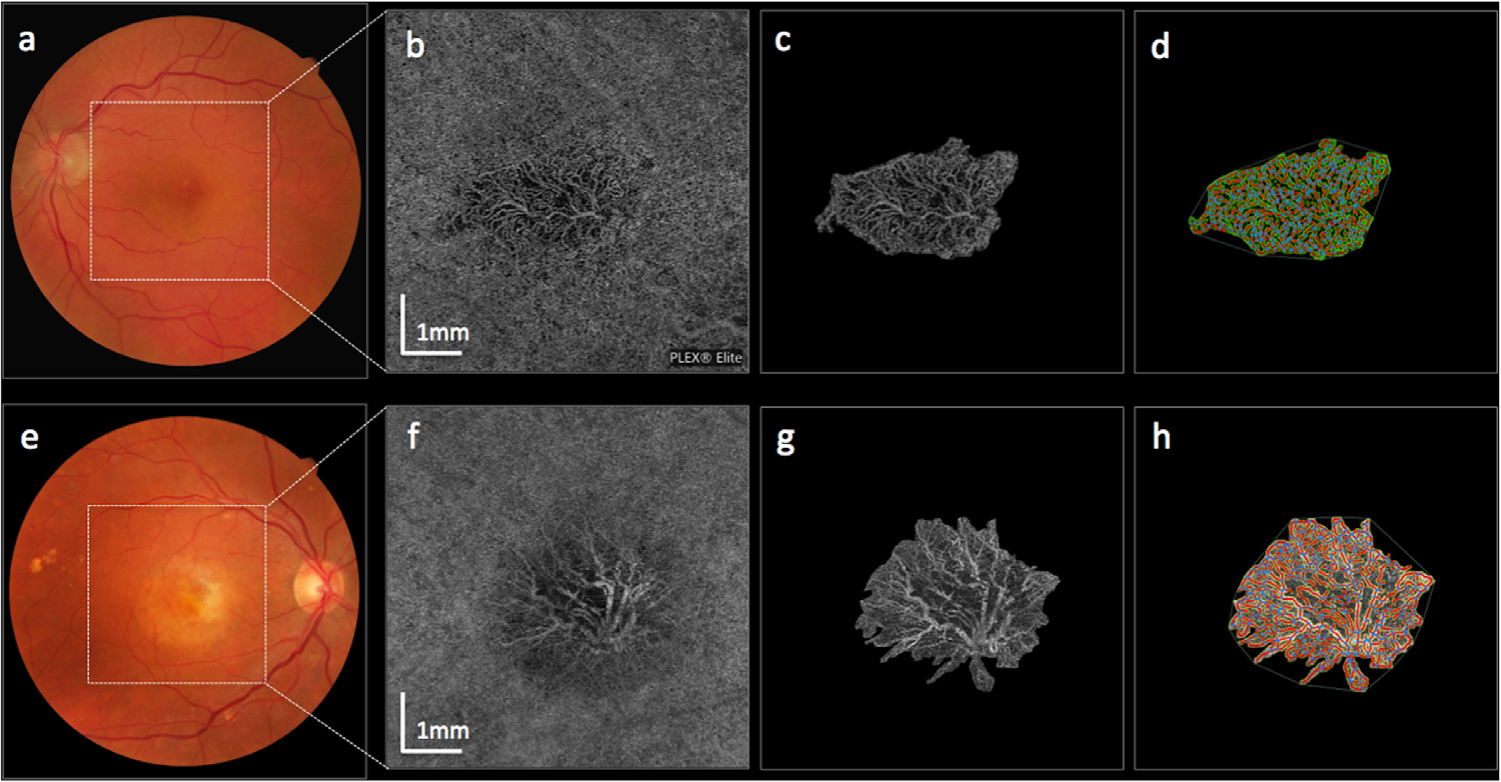
Color fundus photographs (a,e), choriocapillaris en face OCTA slabs (b,f), manually delineated MNV complexes (c,g) and Angiotool analysis results (d, h) are displayed. The top row (a-d) shows a typical example of a MNV lesion without SF. The bottom row (e-h) shows a typical case of a MNV lesion with SF.

Fifty eyes were included in the quantitative analysis, 19 eyes (38%) from the SF group and 31 eyes (62%) from the non-SF group. Representative examples from the SF group and non-SF group are presented in Figure 3. Nine eyes in the non-SF group (five eyes with a retinal angiomatous proliferation lesion and four eyes with a type 1 MNV) and one eye in the SF group (mixed type MNV) could not be evaluated quantitatively. Table 2 provides details about quantitative MNV parameters. The GVC and GLD were larger in the SF group than in the non-SF group (*P* = 0.001 and *P* = 0.042). Furthermore, a larger total lesion area (*P* = 0.026) and a larger total vessel area (*P* = 0.037) was observed in the SF group. The total number of junctions (*P* = 0.025), total vessel length (*P* =0.027) and total number of endpoints (*P* = 0.007) was also larger in the SF group than in the non-SF group, as was endpoint density (*P* = 0.047).

**Table 2.** Quantitative Microvascular Parameters Assessed By Optical Coherence Tomography Angiography

	No subretinal Fibrosis	Subretinal Fibrosis	
Quantitative MNV Features	(n = 31)	(n = 19)	*P* Value
Greatest vascular caliber (µm), median (min-max)	109 (30–229)	161 (67–268)	0.001^*^
Greatest linear diameter (µm), median (min-max)	2366 (415–4190)	3010 (1594–5518)	0.042^*^
Lesion area (mm^2^)	3.62 (0.10–9.80)	4.89 (1.33–17.42)	0.026^*^
Vessel area (mm^2^)	1.57 (0.07–4.82)	2.16 (0.87–7.35)	0.037^*^
Vessels percentage area (%)	48.90 (20.12–71.26)	44.91 (31.72–65.05)	0.250
Number of junctions (n)	156 (4–514)	242 (74–866)	0.025^*^
Total vessel length (mm)	32.88 (1.23–94.11)	43.83 (14.47–161.05)	0.027^*^
Vessel length density (mm/mm^2^)	19.36 (15.74–23.79)	20.46 (16.68–21.99)	0.242
Total number of endpoints (n)	51 (2–215)	96 (9–370)	0.007^*^
Mean lacunarity	0.19 (0.11–0.54)	0.15 (0.09–0.37)	0.250
Junction density (n/mm)	4.98 (3.25–6.92)	5.12 (3.91–6.33)	0.332
Endpoint density (n/mm)	1.41 (0.66–3.27)	1.76 (0.62–3.10)	0.047^*^

Independent samples Mann Whitney U test was used.

* Significant *P* values.

## Discussion

In this cross-sectional observational study, we used gold standard, as well as novel multimodal retinal imaging modalities such as OCTA and PS-OCT, to analyze in detail outer retinal and microvascular differences between eyes with and without SF caused by nAMD. Based on published literature, patients with nAMD have a risk of approximately 35% of developing SF within the first year after diagnosis, further increasing thereafter.^5^^,^^6^^,^^43^ Hence, only patients with a minimum history of 12 months of anti-VEGF treatment were consecutively included to provide a number of participants affected by SF that reflects a real-world scenario. In line with the literature, subretinal MNV (type 2 and mixed type MNV) was common in eyes with SF, whereas in the non-SF group, type 1 MNV was more prevalent (Table 1).^5^^,^^6^^,^^43^^,^^44^ All eyes with SF exhibited a preserved microvascular MNV network. Using quantitative metrics, we found that SF is associated with larger lesions and bigger caliber vessels as shown by the larger GVC and GLD, as well as the larger total lesion area and vessel area. Furthermore, the total number of junctions, total vessel length, total number of endpoints, and endpoint density were larger in the SF group.

We found a difference between eyes with and eyes without SF in GLD, as well as total lesion area and total vessels area, suggesting an association between larger MNV complexes and SF development. Bloch et al.^5^ investigated the baseline characteristics associated with the development of fibrosis within two years in eyes with nAMD under anti-VEGF treatment. They found that an initial MNV area larger than 5 disk areas was associated with a significantly increased risk by a factor of 4.49 over that of a MNV area of 1.5 disk areas or smaller. However, trend testing did not show a significant overall relationship between treatment-naïve MNV area and the development of SF. In our study we did not compare MNV characteristics at baseline but after a median history of anti-VEGF therapy of more than three years, which has most likely changed the configuration and size of the neovascular complex. There was a significant difference in GVC between the two groups, suggesting vascular maturation and abnormalization resulting in thicker caliber vessels with high flow in SF.^45^ The higher total number of vessel junctions and endpoints and the longer total vessel length in the SF group may be expected because of the larger total lesion area and total vessel area. The higher endpoint density indicates a higher number of open-ended vessel segments with fewer connections between the vessels, also indicating vessel abnormalization.^45^

PS-OCT was used as an ancillary imaging modality for SF detection and was a sensitive tool particularly in clinically challenging cases with exudate and yellow-whitish material mimicking SF, as shown in previous studies.^17^^,^^18^^,^^38^ Gräfe et al.^38^ used PS-OCT in a group of patients with CNV clinically diagnosed with suspected fibrosis, doubtful fibrosis, or lesions suspected not to be fibrotic. The authors found that PS-OCT provided additional information and was useful for identification and quantification, particularly in doubtful cases.^38^

In our study ORT was found significantly more often in the SF group than in the non-SF group. Photoreceptors may form ORT in retinal diseases associated with atrophy or degeneration of the RPE, most likely reflecting a survival mechanism of photoreceptors in areas where the photoreceptor support system (i.e., the RPE and choriocapillaris) is missing.^21^ There was no difference in the presence of RPD between the SF and non-SF group in our study population, suggesting no particular role for RPD in fibrosis development. HRF had a higher prevalence in the non-SF group than in the SF group, suggesting more intraretinal RPE migration in clinically active MNV and less intraretinal RPE migration once SF has developed. This finding is in line with histopathologic observations, where intraretinal RPE cells were typically found in eyes with CNV and “entombed” RPE cells were seen in areas of fibrovascular scars.^46^ We previously used PS-OCT to selectively image birefringent fibrous tissue and facilitate differentiation from depolarizing RPE to identify, when nonfibrous subretinal hyperreflective material would turn into SF.^17^^,^^18^ We found that presence of SF was associated with loss or porosity of the RPE layer. We observed continuous RPE in eyes with non-fibrotic subretinal material, but observed only disseminated depolarizing particles in areas of SF, presumably remnants of dissociated RPE. This finding fits well the concept of the epithelial-mesenchymal transition, where, as a result of tissue injury, RPE cells undergo a transformation into a myofibroblast cell form, which contributes to collagen deposition and SF formation.^47^ RPE migration toward a MNV membrane, regularly observed in type 2 MNV, is commonly believed to be beneficial and may serve a barrier function to prevent extension of the neovascular membrane.^48^ Although inhibition of this natural defense mechanism could be detrimental, inhibition of the epithelial-mesenchymal transition may be favorable in eyes with nAMD. The transformation of RPE cells to myofibroblasts has been identified as a possible target for interventional treatment to prevent SF formation and associated functional loss.^10^^,^^49^ A combination of anti-VEGF with anti-fibrotic therapies may be necessary to simultaneously treat MNV exudation, as well as SF formation.

The authors are aware of certain limitations of the study, including the small number of study participants, as well as the cross-sectional study design. The focus of this study was on differences between eyes with and without SF; however, differences between the groups may have been caused by other underlying conditions such as MNV distribution. Another limitation was the fact that nine eyes from the non-SF group and one eye from the SF group had to be excluded from the quantitative analysis, which may have confounded the results. Despite using a long wavelength SS-OCTA device, allowing for better penetration into tissue, the presence of SF may have caused some shadowing on underlying structures. Furthermore, the software for quantitative OCTA analysis, even with parameter optimization, has limitations in accurate identification of vessels with low intensity, which may not have been perfectly segmented. PS-OCT is an imaging modality that is not commercially available, and, hence, interpretation of the additional tissue-specific imaging B-scans may require some experience. An advantage of the use of PS-OCT, however, was the recently implemented fibrosis detection algorithm, which objectively and automatically identifies SF. Positive aspects include a standardized examination protocol, as well as the use of novel imaging methods such as OCTA or PS-OCT, allowing visualization of different aspects of the MNV lesion that would otherwise not be detected using standard modalities.

In conclusion, using multimodal imaging our study demonstrated in vivo microvascular and morphological differences in eyes with MNV secondary to nAMD with and without SF. Eyes with SF tend to have larger MNV lesions with thicker vessels and are often associated with the presence of ORT.
